# Voluntary wheel running attenuates peptidoglycan–polysaccharide‐induced inflammation and preserves skeletal muscle remodeling in male C57BL/6J mice

**DOI:** 10.14814/phy2.70916

**Published:** 2026-05-18

**Authors:** Ryoga Kinoshita, Tatsuhiro Yamaguchi, Satoru Ato, Karina Kouzaki, Yuki Tamura, Koichi Nakazato

**Affiliations:** ^1^ Nippon Sport Science University Tokyo Japan; ^2^ The University of Tokyo Tokyo Japan; ^3^ Japan Society for the Promotion of Science Tokyo Japan

**Keywords:** chronic systemic inflammation, exercise, skeletal muscle

## Abstract

Chronic systemic inflammation (CSI) is associated with skeletal muscle dysfunction and may impair adaptive responses. This study investigated whether skeletal muscle responses to voluntary wheel running (VWR) are maintained following exposure to CSI. Twelve‐week‐old male C57BL/6J mice were assigned to Saline+Sedentary, Peptidoglycan–Polysaccharide (PG‐PS) + Sedentary, Saline+Exercise, and PG‐PS + Exercise groups (*n* = 12 per group), with PG‐PS used to induce CSI. Following 3 weeks of intervention, blood and lower‐leg skeletal muscles were collected. Total running distance did not differ between exercise groups. PG‐PS administration increased serum tumor necrosis factor‐α and interleukin‐1β levels in sedentary mice, whereas these levels were attenuated in VWR mice. Under sedentary conditions, soleus muscle weight and fiber cross‐sectional area (CSA) were lower in PG‐PS‐treated mice. Exercise was associated with higher soleus muscle mass and fiber CSA in both saline‐ and PG‐PS‐treated mice. PG‐PS increased 4‐hydroxynonenal levels in the soleus muscle, whereas VWR was associated with lower levels. VWR markedly increased muscle protein synthesis and reduced markers of muscle protein breakdown, indicating enhanced protein turnover independent of PG‐PS treatment. These findings suggest that exercise‐induced increases in muscle mass and fiber size occur independent of PG‐PS treatment. Clinically, whole‐body exercise may represent a physiologically relevant, non‐pharmacological strategy to support skeletal muscle health under chronic inflammatory conditions.

## 1 INTRODUCTION

Chronic systemic inflammation (CSI) is commonly observed in chronic diseases (e.g., cancer, chronic obstructive pulmonary disease, and chronic kidney disease) (Furman et al., 2019). Although the inflammatory response serves a protective function in tissues through the immune system, unresolved acute inflammation results in chronic low‐grade inflammation (Baylis et al., 2013; Cevenini et al., 2013).

CSI induces skeletal muscle atrophy (Doucet et al., 2007; Kinoshita et al., 2025; Lee et al., 2017). Inflammatory cytokines, such as tumor necrosis factor‐α (TNF‐α), interleukin‐1β (IL‐1β), together with reactive oxygen species (ROS) generated during CSI, suppress muscle protein synthesis (MPS) and enhance muscle protein breakdown (MPB) (Beals et al., 2019; Huang et al., 2017). These alterations impair skeletal muscle remodeling and contribute to reduced physical function.

Various chemical components can induce systemic inflammation in animal models. Although lipopolysaccharide (LPS) is widely used, it primarily induces acute and severe inflammatory responses (Watts et al., 2020). In contrast, peptidoglycan–polysaccharide (PG‐PS), a component of gram‐positive bacteria, produces sustained low‐grade systemic inflammation following a single administration. PG‐PS induces CSI and skeletal muscle atrophy in male C57BL/6J mice, rendering it a suitable model for investigating CSI‐related muscle alterations (Kinoshita et al., 2025).

Exercise is an effective approach to prevent and counteract skeletal muscle atrophy. Among exercise modalities, those that maintain or increase muscle mass are considered appropriate interventions. Electrical stimulation, ladder climbing, and voluntary wheel running (VWR) in mice are commonly used models. However, under CSI exposure, localized muscle contractions caused by electrical stimulation fail to suppress inflammation and elicit only limited hypertrophic effects. In contrast, combining electrical stimulation with an anti‐inflammatory diet attenuates the inhibitory effect of CSI on muscle hypertrophy (Sumi et al., 2020). These findings suggest that suppression of systemic inflammation may be required to preserve training‐induced muscle adaptation under CSI conditions.

VWR is of particular interest because it induces muscle hypertrophy and has been associated with anti‐inflammatory effects (Egawa et al., 2022; Ikeda et al., 2006; Peppler et al., 2016). Given these properties, unlike localized muscle contractions, whole‐body voluntary exercise may preserve skeletal muscle remodeling by attenuating systemic inflammation. However, whether voluntary whole‐body exercise can maintain skeletal muscle remodeling following induction of CSI has not been directly investigated. We therefore hypothesized that VWR would reduce inflammatory and oxidative stress and thereby preserve skeletal muscle hypertrophy and protein turnover responses in mice treated with PG‐PS.

## 2 MATERIALS AND METHODS

### 2.1 Ethical approval

The Animal Experimental Committee of the Nippon Sport Science University (approval no. 021‐A03) approved all animal experiments.

The investigators understand and comply with the ethical checklist of the experimental physiology and regulations of the fundamental guidelines for proper conduct of animal experiments and related activities in academic research institutions published by the Ministry of Education, Culture, Sports, Science, and Technology, Japan (No. 71, 2006).

### 2.2 Materials

The 10S fraction of PG‐PS was purchased from Becton Dickinson (210,866; NJ, USA).

### 2.3 Animals and experimental protocols

Twelve‐week‐old male C57BL/6J mice (Body weight [BW]: 25 ± 3 g) (CREA Japan, Tokyo, Japan) were used in this study. Mice were housed individually in cages maintained at 23°C under a 12:12 h light–dark cycle. They were provided a standard rodent solid diet (CE‐2; CREA Japan) and had ad libitum access to water during acclimatization for at least 1 week.

Subsequently, the mice were assigned to four groups: Saline+Sedentary, PG‐PS + Sedentary, Saline+Exercise, and PG‐PS + Exercise (each group: *n* = 12). We administered a single intraperitoneal injection of PG‐PS or saline (50 μg/g BW).

Mice in the Exercise group were housed in cages (21.58 × 31.68 × 15 cm) equipped with a running wheel assembly (7 × 7 × 6 cm; Merquest Co., Ltd., CIF‐4S, Toyama, Japan). The animals had unrestricted wheel access throughout the intervention period, and running distance was recorded at 1‐min intervals to calculate total distance. Three weeks after PG‐PS administration, the mice were fasted overnight, and blood was collected from the heart under isoflurane anesthesia. Then, cervical dislocation was performed to minimize pain or distress, followed by tissue dissection. The wet weights of the lower hind limb muscles (gastrocnemius, plantaris, and soleus) were recorded. Cross‐sectional area (CSA) samples were embedded in Tissue‐Tek O.C.T. Compound (4583; Sakura Finetek, Tokyo, Japan) and stored at −80°C. Other tissues were rapidly frozen in liquid nitrogen within 15 min of cervical dislocation and stored at −80°C until analysis.

### 2.4 Cytokine measurement

Inflammatory cytokine levels in heart‐derived serum were quantified using enzyme‐linked immunosorbent assay (ELISA) kits according to manufacturer's protocols (TNF‐α: MTA00B‐1; IL‐1β: MLB00C‐1; R&D Systems, Minneapolis, MN, USA). Each blood sample was analyzed twice.

### 2.5 Determination of muscle fiber size and type

Immunohistochemistry for myosin heavy chain isoforms was performed as described by Yamaguchi et al. (Yamaguchi et al., 2025). Cross‐sections (10 μm thick) of the soleus muscle were prepared using a cryostat (CM1860; Leica Biosystems, Wetzlar, Germany) at −20°C. Sections were blocked with 4% bovine serum albumin (BSA) blocking buffer (015‐21,274; Fujifilm Wako Pure Chemical Corporation, Osaka, Japan) for 1 h at 25°C and incubated overnight at 4°C with primary antibodies diluted in BSA blocking buffer: rabbit immunoglobulin G (IgG) polyclonal anti‐laminin antibody (1:1000; L9393; Sigma‐Aldrich, St. Louis, MO, USA), mouse IgG2b polyclonal anti‐myosin heavy chain type I (1:500 dilution; BA‐D5‐s; DSHB, Iowa USA), and IgG1 polyclonal anti‐myosin heavy chain type IIa (1:500 dilution; SC‐71‐s; DSHB). After three 5‐min washes in 0.1 M phosphate buffer (PB; pH 7.4), the sections were incubated overnight in the dark at 4°C with secondary antibodies diluted in BSA blocking buffer: Alexa Fluor™ Plus 647 goat anti‐rabbit IgG H + L (1:1000; A32733; Invitrogen, Waltham, MA, USA), Alexa Fluor 555 goat anti‐mouse IgG2b (1:1000; A21147; Invitrogen), and Alexa Fluor 488 goat anti‐mouse IgG1 (1:1000; A21121; Invitrogen). After washing in 0.1 M PB, the sections were mounted on slides with coverslips using Fluoro‐KEEPER Antifade reagent (12593‐64; Nacalai Tesque, Kyoto, Japan). Muscle cross‐sectional images were acquired using a confocal microscope (FV3000, Olympus, Japan) with a ×10 objective lens. Myofibre size and fiber type distribution were analyzed using the MyoVision software following the method described by Yuan Wen et al. (Yuan Wen et al., 2018). Fibers were classified as either type I (stained with Alexa Fluor 555), type IIa (stained with Alexa Fluor488) or type IIb/IIx (not stained). The average number of analyzed muscle fibers per mouse was as follows: Saline+Sedentary, 394 ± 45; PG‐PS + Sedentary, 421 ± 72; Saline+Exercise, 374 ± 14; and PG‐PS + Exercise, 402 ± 51.

### 2.6 Western blotting

Protein detection, using western blotting, was performed as described by Kasai et al. (Kasai et al., 2022). The gastrocnemius and soleus muscles were homogenized in radioimmunoprecipitation buffer (188–02453; Fujifilm Wako Pure Chemical Corporation) containing protease and phosphatase inhibitor cocktails (169‐26063/167‐24381; Fujifilm Wako Pure Chemical Corporation). The homogenates were centrifuged at 15,000 × *g* and 4°C for 15 min, and the supernatants were collected. Protein concentrations in the lysates were determined using a bicinchoninic acid (BCA) protein assay kit (23,225; Pierce BCA Protein Assay Kit; Thermo Fisher Scientific, Waltham, MA, USA). Equal amounts (5 μg) of protein were separated by sodium dodecyl sulphate–polyacrylamide gel electrophoresis (10%, 12% [w/v]; TGX polyacrylamide gel, 161‐0173,1610175 Bio‐Rad, California, USA) and transferred to polyvinylidene difluoride membranes (IPVH00010; Immobilon‐P Membrane, EMD Millipore, Billerica, MA, USA). Protein transfer was verified using Ponceau S staining (33427.01; SERVA Electrophoresis, Heidelberg, Germany). The membranes were blocked with a blocking reagent (NYPBR01; Toyobo Company, Osaka, Japan) for 1 h at 25°C and incubated overnight with primary antibodies diluted in Reagent 1 (NKB‐101; Toyobo Company).

The following antibodies were used: anti‐voltage‐dependent anion channel (VDAC; 12,454; Cell Signaling Technology [CST], Massachusett, USA), cytochrome c oxidase IV (COXIV; 4844; CST), 4‐hydroxynonenal (4‐HNE; MHN‐020P; Japan Institute for the Control of Aging, Shizuoka, Japan), puromycin (MABE343; EMD Millipore, Massachusetts, USA), phospho‐p70S6K (9234), total p70S6K (9202), phospho‐ribosomal protein S6 (rpS6; 2215), total rpS6 (2217), phospho‐4E‐binding protein 1 (4E‐BP1; 9459), and total 4E‐BP1 (9452; all from CST). All primary antibodies were diluted 1:1000. Following incubation, the membranes were washed thrice for 5 min each in Tris‐buffered saline containing 0.01% Tween 20 (TBST; T9142; Takara Bio, Shiga, Japan) and incubated for 1 h at 25°C with secondary antibodies (anti‐rabbit or anti‐mouse IgG horseradish peroxidase (HRP)‐linked antibody; 7074/7076; CST) diluted in Reagent 2 (NKB‐101; Toyobo Company). After incubation, the membranes were washed with TBST, and specific protein bands were visualized using a chemiluminescent reagent (34579; SuperSignal West Pico Chemiluminescent Substrate; Thermo Fisher Scientific). Carbonylated proteins were detected according to the manufacturer's protocol of the OxyBlot™ Protein Oxidation Detection Kit (S7150; EMD Millipore). The blots were scanned using a CCD imager (170‐8071; ChemiDoc XRS, Bio‐Rad) and quantified using Quantity One software (170‐9600; for Windows, v.5.2.1; Bio‐Rad). Ponceau S signal intensity served as the loading control.

### 2.7 Muscle protein synthesis (MPS)

MPS was assessed using the in vivo SUnSET method (Goodman et al., 2011). Puromycin (0.04 μmol/g BW in saline; 160‐23151; Fujifilm Wako Pure Chemical Corporation) was intraperitoneally injected into mice under isoflurane anesthesia. Overall, 15 min after injection, skeletal muscle and adipose tissues were collected. As described above, western blotting was used to detect puromycin incorporation into the gastrocnemius muscle.

### 2.8 RNA isolation, reverse transcription, and quantitative reverse transcription‐PCR

RNA isolation was performed as described by Tamura et al. (Tamura et al., 2024). The soleus muscle was homogenized on ice in TRIzol reagent (15596018; Thermo Fisher Scientific) and separated into organic and aqueous phases using chloroform. RNA was isolated from the aqueous phase, and purity was measured by spectrophotometry (ND‐ONE‐W; Nanodrop One, Thermo Fisher Scientific). Total RNA (1.0 μg) was reverse‐transcribed using a commercial kit (FSQ‐201; Toyobo Company) with random hexamer primers. Gene expression was quantified using Thunderbird SYBR qPCR Mix (QPS‐201; Toyobo Company) and a thermal cycler with an optical reaction module (CFX96 Touch; Bio‐Rad). Glyceraldehyde‐3‐phosphate dehydrogenase (GAPDH) was used as the housekeeping gene, as its expression remained stable across all groups. The primer sequences used were as follows:

Muscle atrophy F‐box (Atrogin‐1)‐Fwd: 5′‐AAGGCTGTTGGAGCTGATAGCA‐3′,

Atrogin‐1‐Rev: 5′‐CACCCACATGTTAATGTTGCCC‐3′,

Muscle RING finger 1 (MuRF‐1)‐Fwd: 5′‐TGCCTGGAGATGTTTACCAAGC‐3′,

MuRF‐1‐Rev: 5′‐AAACGACCTCCAGACATGGACA‐3′,

GAPDH‐Fwd: 5′‐AACTTTGGCATTGTGGAAGG‐3′,

GAPDH‐Rev: 5′‐ACACATTGGGGGTAGGAACA‐3′.

### 2.9 Statistical analysis

All values are expressed as mean ± standard deviation. Comparisons between two groups were performed using Student's unpaired *t*‐test. Two‐factor analyses were conducted using a two‐way analysis of variance. When an interaction was identified, post‐hoc comparisons were performed using the Bonferroni test. If no interaction was detected, the main effect of each factor was analyzed. Statistical significance was set at *p* < 0.05. All analyses were conducted using GraphPad Prism 10 (Mac version 10.3.0; GraphPad Software, CA, USA).

## 3 RESULTS

### 3.1 PG‐PS transiently reduced daily running distance but did not alter total running distance

**FIGURE 1:**
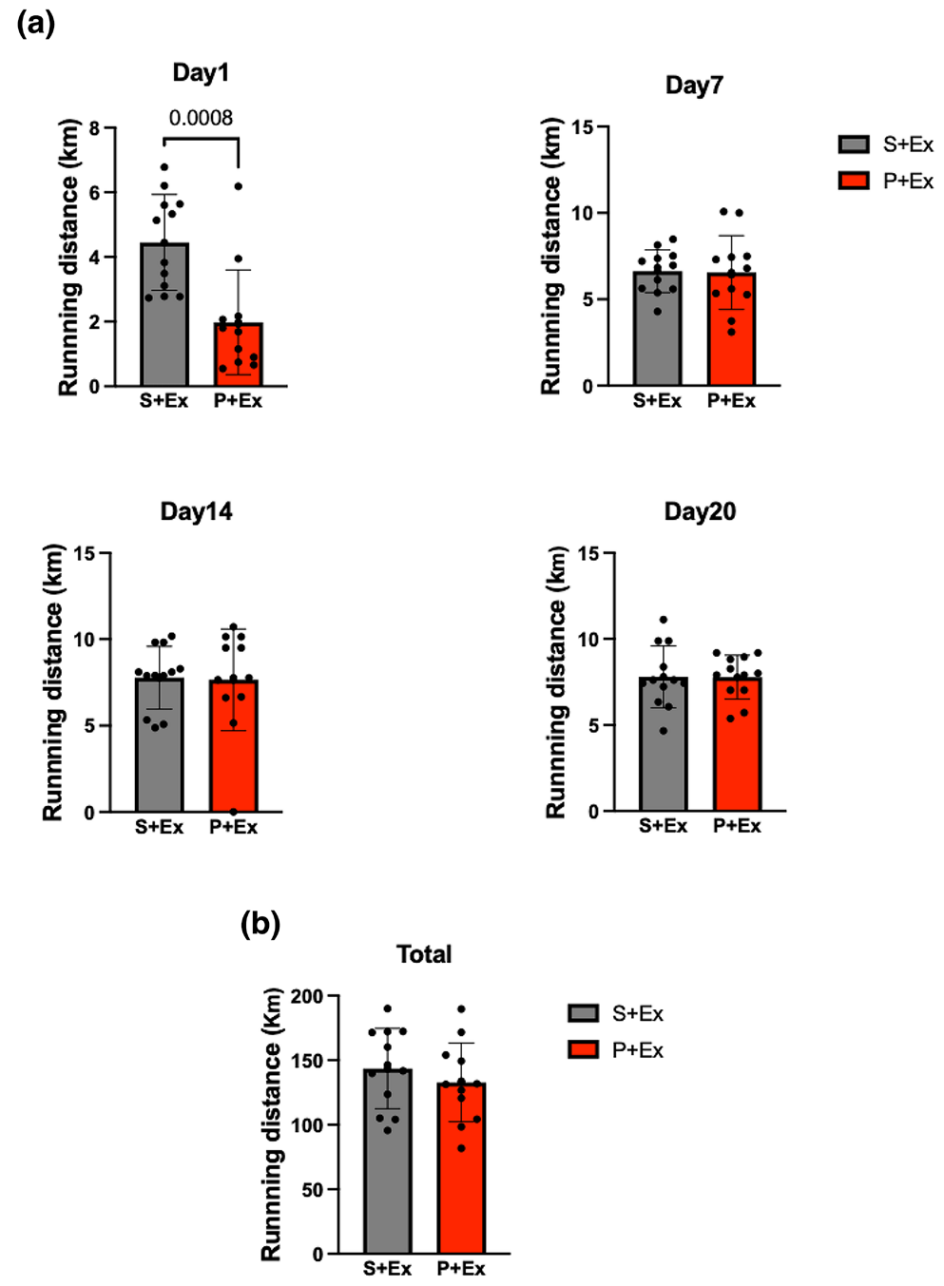
Although a significant reduction was observed in the PG‐PS + Ex group on Day 1, running distance did not differ between the Saline + Ex and PG‐PS + Ex groups overall. (a) Daily running distances recorded on Days 1, 7, 14, and 20 (Saline + Ex group, *n* = 12; PG‐PS + Ex group, *n* = 12). (b) Total running distance (Saline + Ex group, *n* = 12; PG‐PS + Ex group, *n* = 12). Data are shown as mean ± SD. Student's unpaired *t*‐test. Ex, exercise; PG‐PS, peptidoglycan‐polysaccharide; SD, standard deviation.

Daily and total running distances were assessed. A significant reduction in daily running distance was observed in the PG‐PS‐treated group on Day 1 (*p* = 0.0008, Figure 1a). In contrast, the total running distance did not significantly differ between the Saline + Exercise and PG‐PS + Exercise groups (*p* = 0.40, Figure 1b).

### 3.2 PG‐PS elevated serum TNF‐α and IL‐1β levels in sedentary mice, which were attenuated by voluntary wheel running (VWR)

**FIGURE 2:**
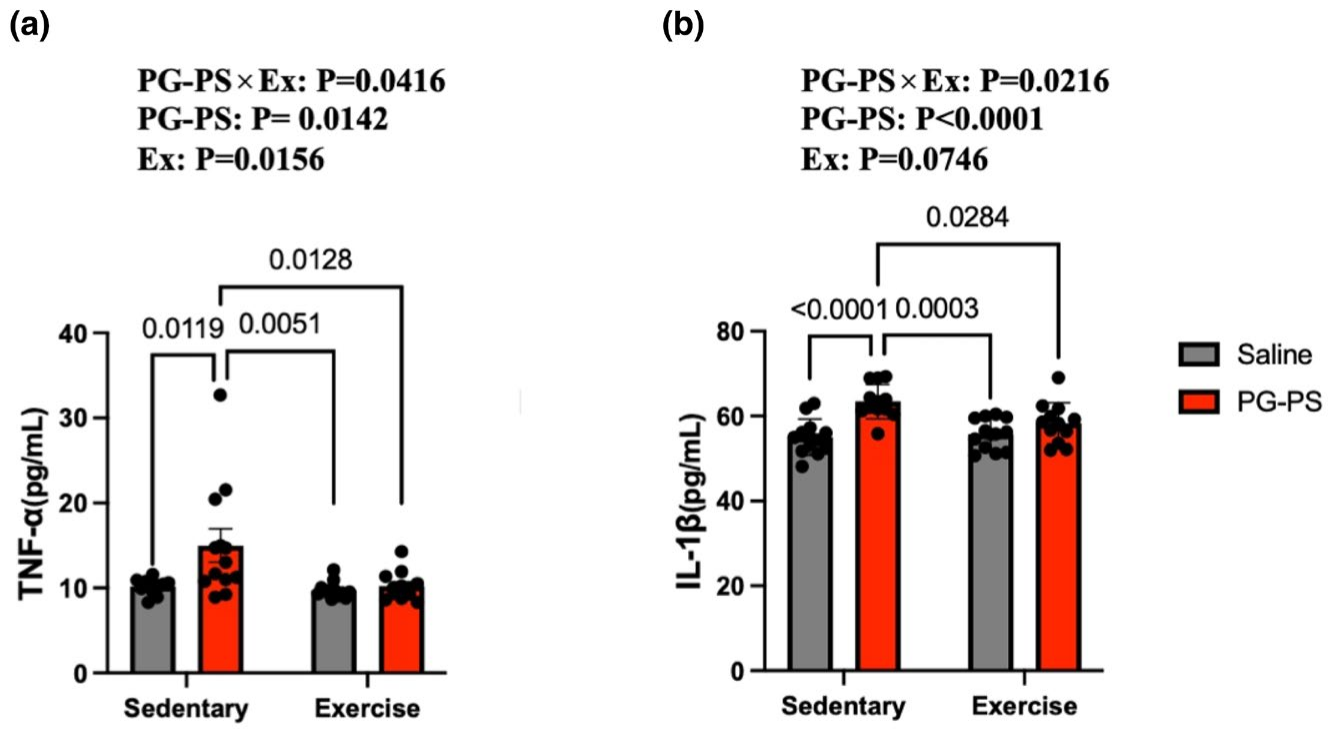
Inflammatory cytokines were increased after PG‐PS treatment, and voluntary wheel running (VWR) attenuated their expression. (a) Concentration of tumor necrosis factor‐α (TNF‐α) in the serum (Saline+Sed group, *n* = 12; PG‐PS + Sed group, *n* = 12; Saline+Ex group, *n* = 12; PG‐PS + Ex group, *n* = 12). (b) Concentration of interleukin‐1β (IL‐1β) in the serum (Saline+Sed group, *n* = 12; PG‐PS + Sed group, *n* = 12; Saline+Ex group, *n* = 12; PG‐PS + Ex group, *n* = 12). Data are shown as mean ± SD. A two‐way ANOVA was performed, followed by a post‐hoc test. Sed, sedentary.

Serum concentrations of TNF‐α and IL‐1β were quantified using ELISA. The PG‐PS + Sedentary group showed significantly higher levels of both cytokines than the Saline+Sedentary group. In contrast, the PG‐PS + Exercise group exhibited a significant decrease in the levels of these cytokines (*p* = 0.01, *p* < 0.0001, *p* = 0.01, and *p* = 0.02, respectively, Figure 2a,b).

### 3.3 Voluntary wheel running (VWR) increased food intake without changing body weight (BW)

**FIGURE 3:**
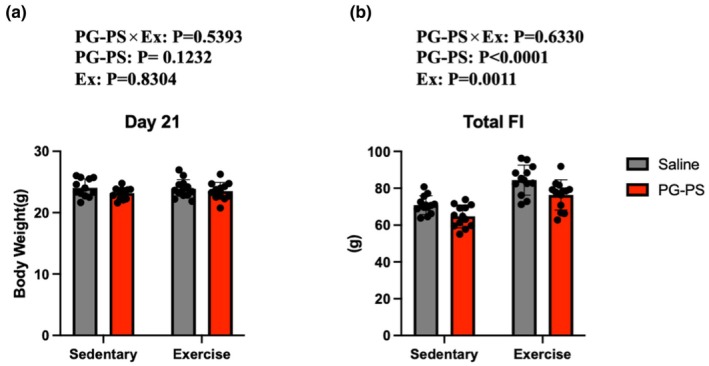
VWR increased food intake in mice. (a) Body weight in male mice (Saline+Sed group, *n* = 12; PG‐PS + Sed group, *n* = 12; Saline+Ex group, *n* = 12; PG‐PS + Ex group, *n* = 12). (b) Total food intake in male mice (Saline+Sed group, *n* = 12; PG‐PS + Sed group, *n* = 12; Saline+Ex group, *n* = 12; PG‐PS + Ex group, *n* = 12). Data are shown as mean ± SD. A two‐way ANOVA was performed.

BW and food intake were analyzed. BW did not differ among the groups (*p* = 0.53, Figure 3a), whereas total food intake was significantly affected by both PG‐PS administration and exercise, with lower values in PG‐PS‐treated mice and higher values in exercised mice overall (*p* < 0.0001, *p* = 0.001, respectively, Figure 3b).

### 3.4 Voluntary wheel running increased muscle weight in both saline‐ and PG‐PS‐treated mice

**FIGURE 4:**
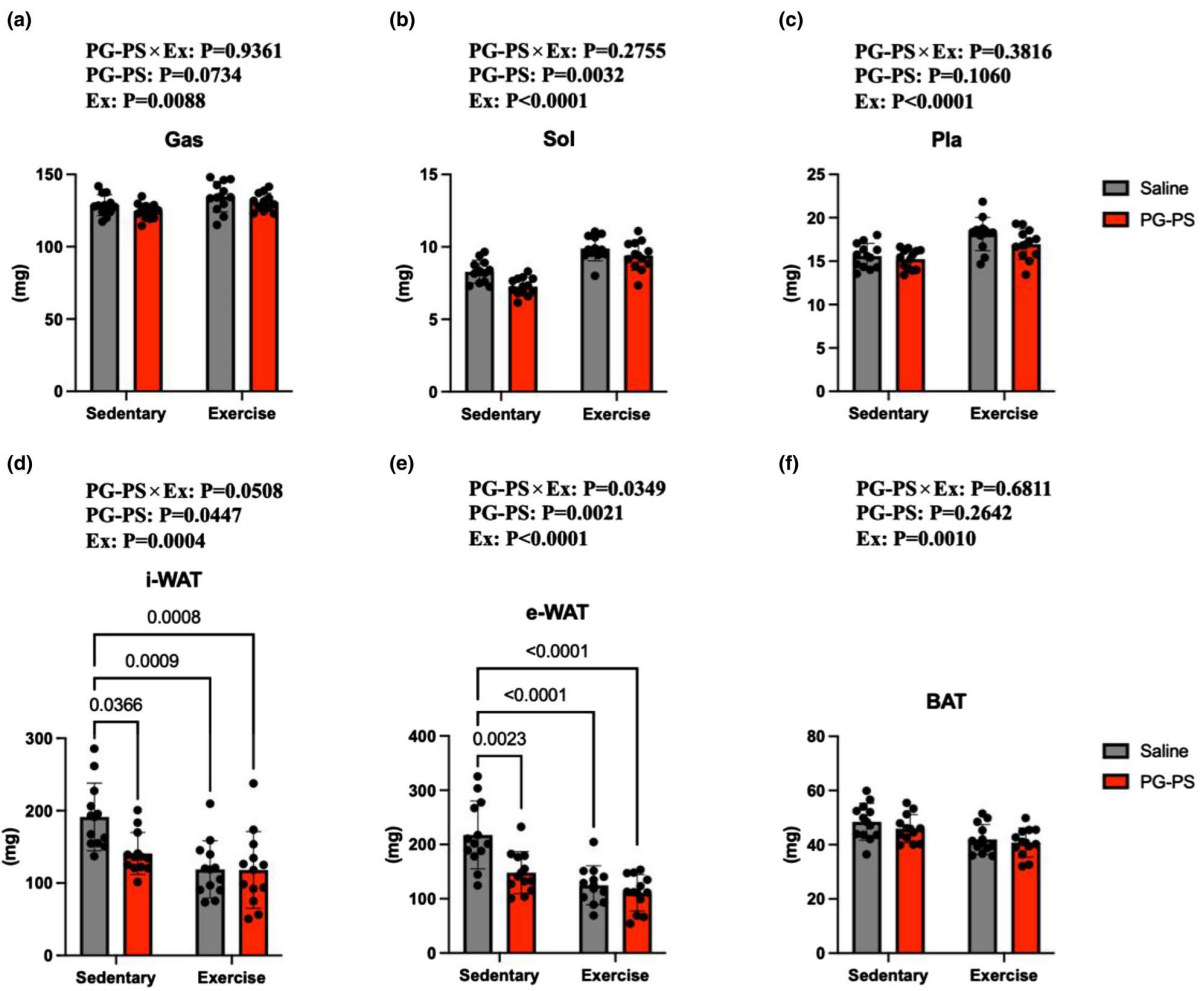
PG‐PS was associated with lower soleus muscle weight, whereas VWR increased muscle weight in the lower leg, independent of PG‐PS treatment. White adipose tissue (WAT) weight was decreased by PG‐PS and exercise. (a) Gastrocnemius muscle weight (Saline+Sed group, *n* = 12; PG‐PS + Sed group, *n* = 12; Saline+Ex group, *n* = 12; PG‐PS + Ex group, *n* = 12). (b) Soleus muscle weight (Saline+Sed group, *n* = 12; PG‐PS + Sed group, *n* = 12; Saline+Ex group, *n* = 12; PG‐PS + Ex group, *n* = 12). (c) Plantaris muscle weight (Saline+Sed group, *n* = 12; PG‐PS + Sed group, *n* = 12; Saline+Ex group, *n* = 12; PG‐PS + Ex group, *n* = 12). (d) Inguinal WAT weight (Saline+Sed group, *n* = 12; PG‐PS + Sed group, *n* = 12; Saline+Ex group, *n* = 12; PG‐PS + Ex group, *n* = 12). (e) Epididymal WAT weight (Saline+Sed group, *n* = 12; PG‐PS + Sed group, *n* = 12; Saline+Ex group, *n* = 12; PG‐PS + Ex group, *n* = 12). (f) Neck brown adipose tissue (BAT) weight (Saline+Sed group, *n* = 12; PG‐PS + Sed group, *n* = 12; Saline+Ex group, *n* = 12; PG‐PS + Ex group, *n* = 12). Data are shown as mean ± SD. A two‐way ANOVA was performed, followed by a post‐hoc test.

Weights of skeletal muscle and adipose tissue were measured. The inguinal white adipose tissue (i‐WAT) and epididymal white adipose tissue (e‐WAT) masses were significantly lower in the PG‐PS + Sedentary group than in the Saline + Sedentary group (*p* = 0.03 and *p* = 0.002, respectively, Figure 4d,e). Furthermore, the exercise groups exhibited a significant reduction in WAT mass (*p* < 0.0009, *p* < 0.0001, respectively,Figure 4d,e). Significant effects of PG‐PS and exercise were observed in the soleus muscle, with lower values in PG‐PS‐treated mice and higher values in exercised mice overall (*p* = 0.003, *p* < 0.0001, respectively, Figure 4b). Also, the impact of exercise was evident in other lower leg muscles (*p* = 0.0088, *p* < 0.0001, respectively, Figure 4a,c). These findings indicate that muscle weight was lower in the PG‐PS + Sedentary group compared with the Saline + Sedentary group. VWR significantly increased muscle weight in both saline‐ and PG‐PS‐treated mice. These results suggest that the exercise‐induced increase in muscle weight was not impaired by PG‐PS treatment.

### 3.5 Voluntary wheel running (VWR) increased soleus muscle fiber CSA without altering fiber‐type composition irrespective of PG‐PS treatment

**FIGURE 5:**
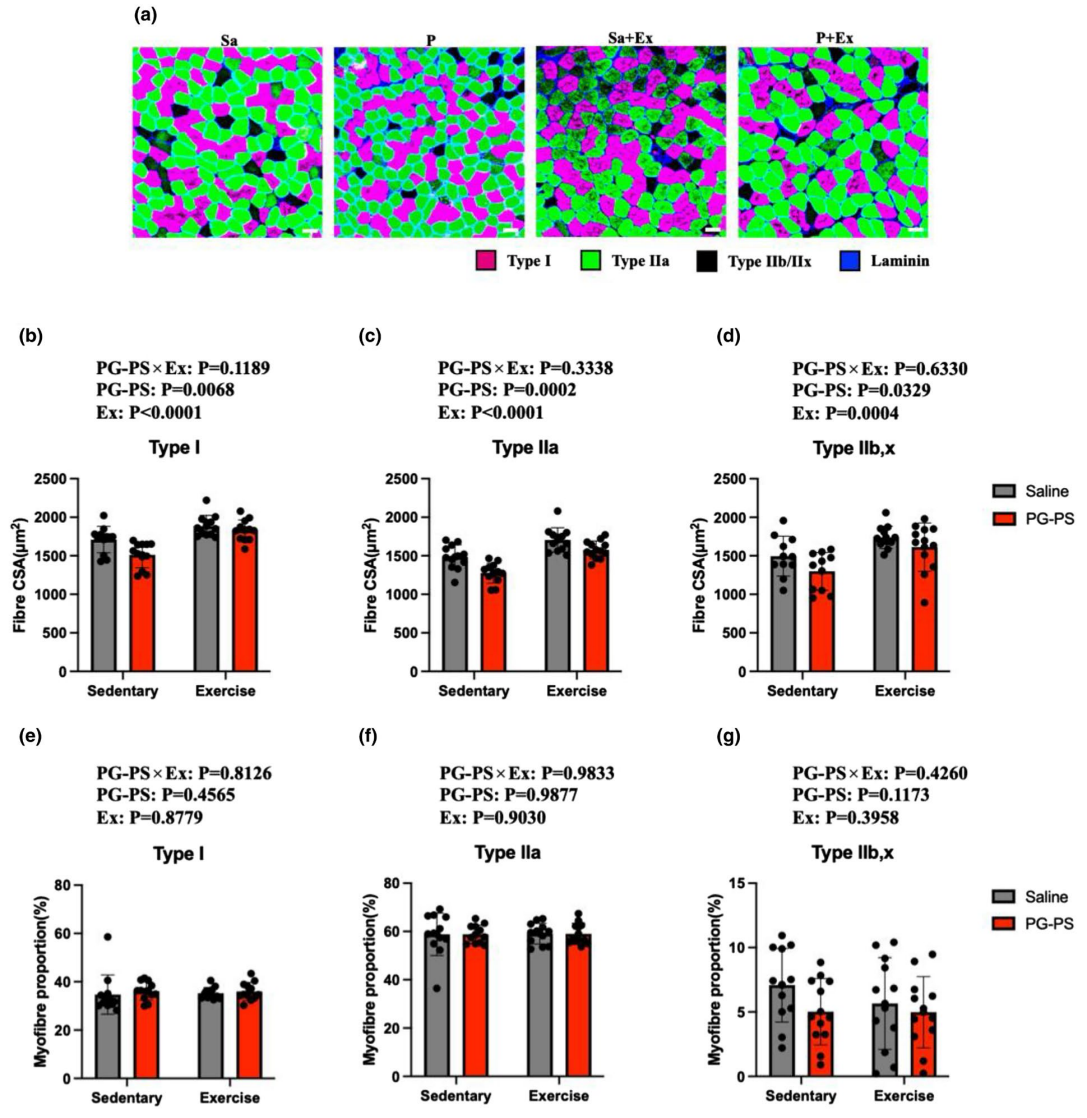
PG‐PS was associated with lower skeletal muscle fiber cross‐sectional area (CSA), whereas VWR increased CSA in the soleus muscle. Laminin was stained in blue, type I fibers in magenta, and type IIa fibers in green. (a) Representative image of the soleus muscle (Scale bar = 100 μm). (b) Type I fiber CSA of soleus muscle (Saline+Sed group, *n* = 12; PG‐PS + Sed group, *n* = 12; Saline+Ex group, *n* = 12; PG‐PS + Ex group, *n* = 12). (c) Type IIa fiber CSA of the soleus muscle (Saline+Sed group, *n* = 12; PG‐PS + Sed group, *n* = 12; Saline+Ex group, *n* = 12; PG‐PS + Ex group, *n* = 12). (d) Type IIb/IIx fiber CSA of the soleus muscle (Saline+Sed group, *n* = 12; PG‐PS + Sed group, *n* = 12; Saline+Ex group, *n* = 12; PG‐PS + Ex group, *n* = 12). (e) Type I fiber proportion of the soleus muscle (Saline+Sed group, *n* = 12; PG‐PS + Sed group, *n* = 12; Saline+Ex group, *n* = 12; PG‐PS + Ex group, *n* = 12). (f) Type IIa fiber proportion of the soleus muscle (Saline+Sed group, *n* = 12; PG‐PS + Sed group, *n* = 12; Saline+Ex group, *n* = 12; PG‐PS + Ex group, *n* = 12). (g) Type IIb/IIx fiber proportion of the soleus muscle (Saline+Sed group, *n* = 12; PG‐PS + Sed group, *n* = 12; Saline+Ex group, *n* = 12; PG‐PS + Ex group, *n* = 12). Data are shown as mean ± SD. A two‐way ANOVA was performed.

We analyzed myofibre composition and size in the soleus muscle to assess skeletal muscle remodeling. Significant effects of PG‐PS and exercise were observed on CSA across all fiber types, with lower values in PG‐PS‐treated mice and higher values in exercised mice overall. (*p* = 0.0001–0.03, Figure 5b–d). However, fiber‐type proportions did not differ significantly among groups (*p* = 0.1173–0.9877, Figure 5e–g). These results showed a trend similar to that observed for muscle wet weight, indicating that VWR‐induced muscle adaptation was not impaired by PG‐PS treatment.

### 3.6 PG‐PS and voluntary wheel running (VWR) did not alter mitochondrial protein expression in the soleus muscle

**FIGURE 6:**
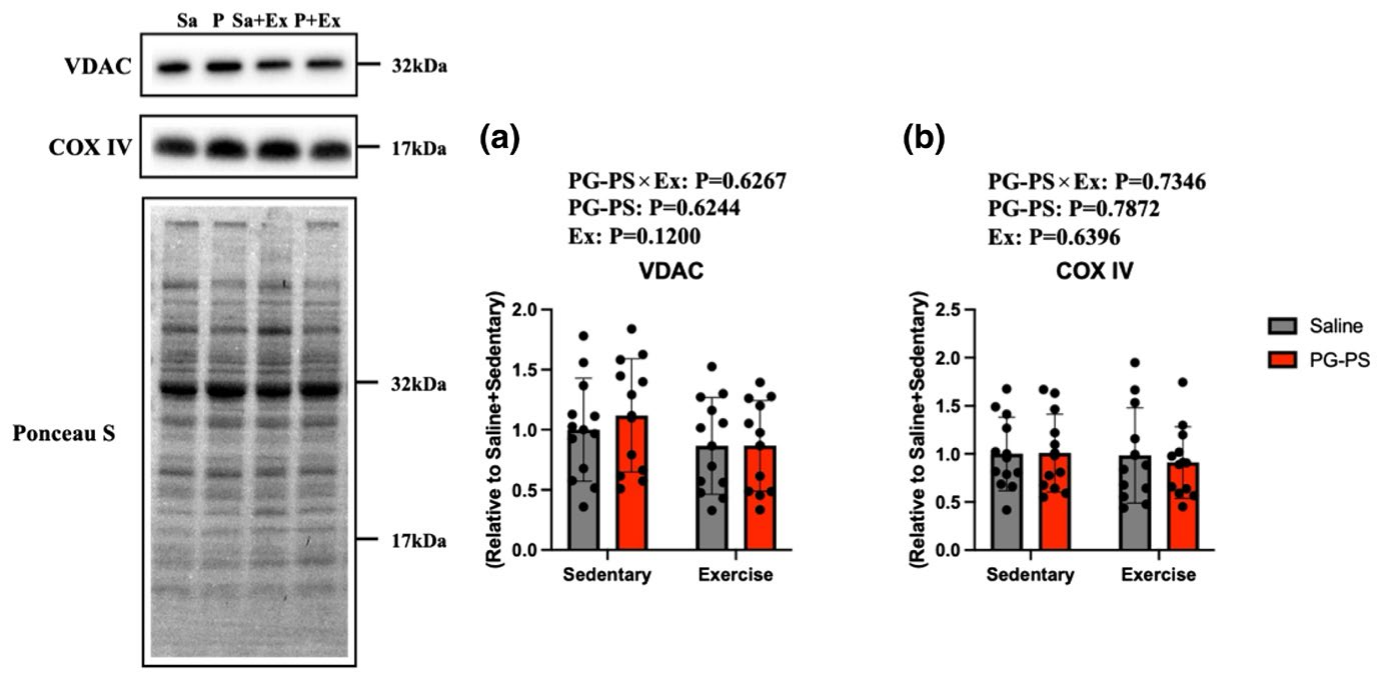
PG‐PS and VWR did not affect the expression of mitochondria‐related proteins in the soleus muscle. (a) Expression of voltage‐dependent anion channel (VDAC) in soleus muscle (Saline+Sed group, *n* = 12; PG‐PS + Sed, *n* = 12; Saline+Ex, *n* = 12; PG‐PS + Ex group, *n* = 12). (b) Expression of Cytochrome c oxidase IV (COX IV) in the soleus muscle (Saline+Sed group, *n* = 12; PG‐PS + Sed group, *n* = 12; Saline+Ex group, *n* = 12; PG‐PS + Ex group, *n* = 12). Data are shown as mean ± SD. A two‐way analysis of variance (ANOVA) was performed.

As chronic systemic inflammation and exercise influence mitochondrial adaptation in skeletal muscle (Tamura et al., 2024; Tang et al., 2013), we examined whether PG‐PS‐induced CSI and VWR affected mitochondrial content in the soleus muscle. Protein expression levels of COX IV and VDAC, which are mitochondrial biomarkers, were examined. Neither protein differed significantly among groups (*p* = 0.12–0.7872, Figure 6a,b), indicating that PG‐PS treatment and VWR did not alter mitochondrial content or muscle fiber type composition.

### 3.7 PG‐PS increased 4‐HNE levels in muscle, which were partially reduced by voluntary wheel running (VWR)

**FIGURE 7:**
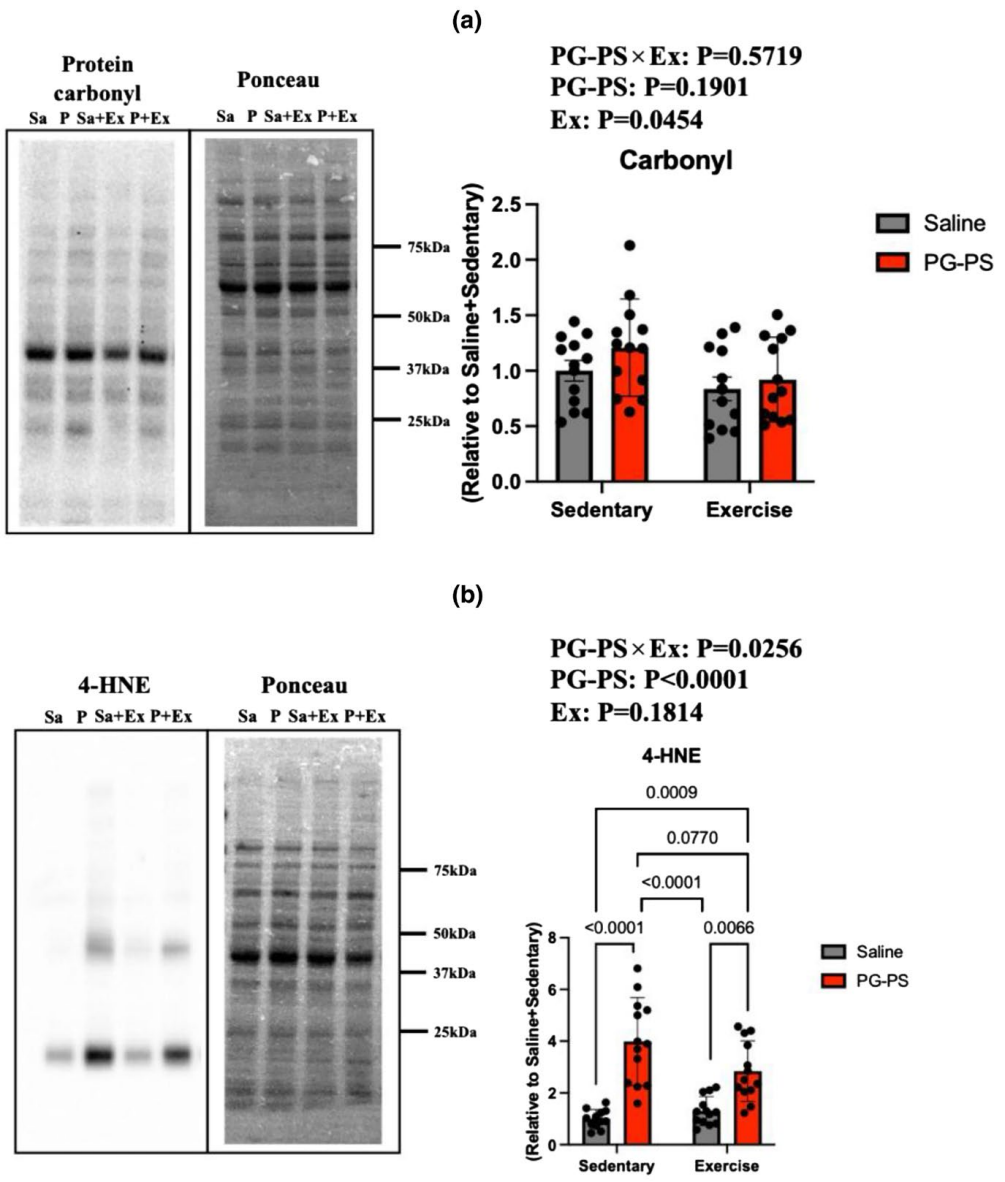
PG‐PS increased the expression of reactive oxygen species (ROS)‐related proteins, whereas VWR attenuated these proteins. (a) Expression of carbonyl protein in the soleus muscle (Saline+Sed group, *n* = 12; PG‐PS + Sed group, *n* = 12; Saline+Ex group, *n* = 12; PG‐PS + Ex group, *n* = 12). (b) Expression of 4‐hydroxynonenal (4‐HNE) in the soleus muscle (Saline+Sed group, *n* = 12; PG‐PS + Sed group, *n* = 12; Saline+Ex group, *n* = 12; PG‐PS + Ex group, *n* = 12). Data are shown as mean ± SD. A two‐way ANOVA was performed, followed by a post‐hoc test.

To evaluate oxidative stress, ROS‐related proteins in the soleus muscle were analyzed. Carbonyl protein levels were used to determine the main effect of exercise on soleus muscle (*p* = 0.0454, Figure 7a). PG‐PS treatment significantly increased 4‐HNE levels; however, this increase was attenuated by VWR in PG‐PS‐treated mice (*p* < 0.0001, *p* = 0.077, respectively, Figure 7b). Thus, PG‐PS administration consistently elevated lipid oxidation in the muscle, whereas VWR partially suppressed it.

### 3.8 Voluntary wheel running (VWR) increased MPS independent of PG‐PS treatment

**FIGURE 8:**
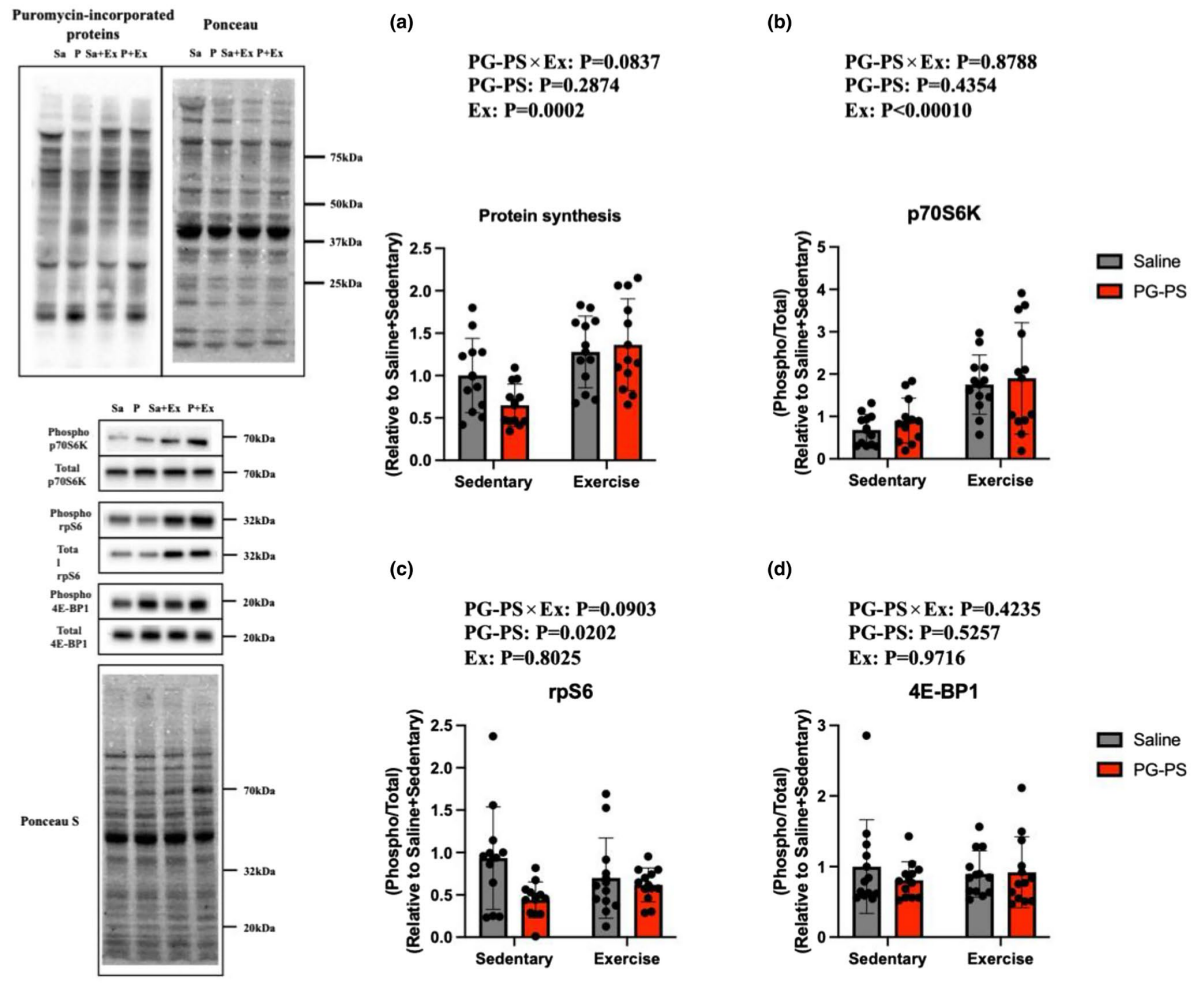
PG‐PS treatment decreased ribosomal protein S6 (rpS6) phosphorylation, whereas VWR increased p70S6K phosphorylation. (a) Puromycin‐incorporated protein in the soleus muscle (Saline+Sed group, *n* = 12; PG‐PS + Sed group, *n* = 12; Saline+Ex group, *n* = 12; PG‐PS + Ex group, *n* = 12). (b) Phosphorylated per total p70 ribosomal S6 kinase (p70S6K) in the soleus muscle (Saline+Sed group, *n* = 12; PG‐PS + Sed group, *n* = 12; Saline+Ex group, *n* = 12; PG‐PS + Ex group, *n* = 12). (c) Phosphorylated per total rpS6 in the soleus muscle (Saline+Sed group, *n* = 12; PG‐PS + Sed group, *n* = 12; Saline+Ex group, *n* = 12; PG‐PS + Ex group, *n* = 12). (d) Phosphorylated per total 4E‐binding protein 1 (4E‐BP1) in the soleus muscle (Saline+Sed group, *n* = 12; PG‐PS + Sed group, *n* = 12; Saline+Ex group, *n* = 12; PG‐PS + Ex group, *n* = 12). Data are shown as mean ± SD. A two‐way ANOVA was performed.

Skeletal muscle protein synthesis and its upstream signaling pathways were evaluated in the soleus muscle. Puromycin‐labeled proteins, used as indicators of MPS, exhibited the main effect of VWR, with higher values in exercised mice overall. (*p* = 0.0002, Figure 8a). Additionally, the phosphorylation of proteins involved in the mechanistic target of the rapamycin (mTOR)C1 signaling pathway was examined. p70S6K phosphorylation was primarily affected by exercise, with higher values in exercised mice overall (*p* < 0.0001, Figure 8b). In contrast, rpS6 phosphorylation was primarily influenced by PG‐PS, with lower values in PG‐PS‐treated mice overall (*p* = 0.0202, Figure 8c). No significant differences were observed in 4E‐BP1 phosphorylation under either PG‐PS or exercise conditions (*p* = 0.5257, *p* = 0.9716, respectively, Figure 8d). Overall, PG‐PS selectively affected components of the mTORC1 signaling pathway, whereas VWR significantly increased MPS at the time point examined, irrespective of PG‐PS treatment.

### 3.9 Voluntary wheel running (VWR) reduced expression of Atrogin‐1 and MuRF‐1 in the soleus muscle

**FIGURE 9:**
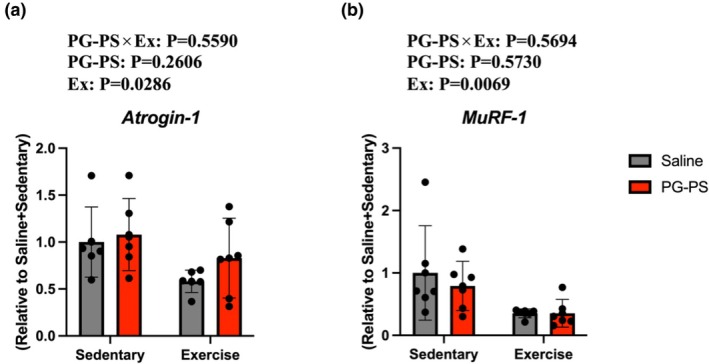
VWR suppressed the mRNA expression of genes involved in muscle protein breakdown (MPB). (a) Muscle atrophy F‐box (Atrogin‐1) mRNA expression in the soleus muscle (Saline+Sed group, *n* = 6; PG‐PS + Sed group, *n* = 6; Saline+Ex group, *n* = 6; PG‐PS + Ex group, *n* = 6). (b) Muscle RING finger 1 (MuRF‐1) mRNA expression in the soleus muscle (Saline+Sed group, *n* = 6; PG‐PS + Sed group, *n* = 6; Saline+Ex group, *n* = 6; PG‐PS + Ex group, *n* = 6). Data are shown as mean ± SD. A two‐way ANOVA was performed.

The expression of proteins related to the ubiquitin–proteasome pathway was analyzed in the soleus muscle. The expression of both Atrogin‐1 and MuRF‐1 was mainly affected by exercise, with lower values in exercised mice overall (*p* = 0.0286, *p* = 0.0069, respectively, Figure 9a,b). These findings suggest that VWR may reduce MPB.

## 4 DISCUSSION

In this study, we used a PG‐PS–induced CSI mouse model to investigate whether VWR‐induced skeletal muscle remodeling is maintained following PG‐PS treatment. The main findings were as follows: (1) PG‐PS increased serum inflammatory cytokine levels in male C57BL/6J mice and was associated with lower soleus muscle weight under sedentary conditions; (2) PG‐PS treatment was associated with increased oxidative stress–related proteins and alterations in components of mTORC1 signaling in the soleus muscle; and (3) inflammatory cytokine levels were attenuated in the PG‐PS + Exercise group, and VWR increased soleus muscle weight in both saline‐ and PG‐PS‐treated mice, suggesting that exercise‐induced increases in muscle mass were not impaired by PG‐PS treatment.

### 4.1 Relationship between inflammation, food intake, and body weight (BW) following CSI

In this study, CSI exposure was confirmed by elevated circulating inflammatory cytokine levels in sedentary PG‐PS–treated mice; however, no significant differences in BW were observed. This finding is consistent with previous studies, performed using pharmacologically induced CSI models, suggesting that inflammatory responses associated with CSI do not necessarily lead to changes in BW (Aries et al., 2023; De La Serre et al., 2015). CSI can cause tissue damage, but such changes are not always reflected in gross outcomes, such as BW. Furthermore, unlike acute inflammation, chronic exposure may induce adaptive responses, potentially improving feeding behavior and alleviating metabolic disturbances. Therefore, future studies should elucidate the underlying mechanisms in greater detail.

Additionally, the CSI + Sedentary group showed reduced total food intake. Previous studies using PG‐PS reported a significant decrease in food intake on Day 3 following administration. This suggests that reduced feeding during the acute inflammation phase may have contributed to the results of the present study (Kinoshita et al., 2025). Although food intake increased in the VWR group, no differences in BW were detected, which may be attributed to the elevated energy expenditure caused by VWR. Future studies should evaluate activity levels in sedentary groups to further clarify these findings.

### 4.2 Inflammatory response and muscle atrophy following exposure to PG‐PS–induced CSI

In this study, PG‐PS significantly increased TNF‐α and IL‐1β levels in the blood, consistent with earlier findings (Kinoshita et al., 2025). Cytokine levels have been shown to correlate with the extent of skeletal muscle atrophy (Sumi et al., 2020). Thus, pro‐inflammatory cytokines may contribute to muscle atrophy in PG‐PS‐induced CSI.

### 4.3 Oxidative stress and MPS

Skeletal muscle mass is maintained by the balance between MPS and MPB (Burd et al., 2009). MPS is primarily regulated by the mTOR signaling pathway (Ogasawara & Suginohara, 2018). mTOR exists in at least two complexes: mTOR complex 1 (mTORC1) and mTORC2 (Chiappinelli et al., 2017). mTORC1 is activated by stimuli such as muscle contraction and nutrient intake, leading to the phosphorylation of downstream targets, including p70S6K, rpS6, and 4E‐BP1, which promote protein synthesis (Drummond et al., 2009). Conversely, MPB is largely regulated by the ubiquitin–proteasome pathway, with the E3 ubiquitin ligases MuRF1 and MAFbx serving as key regulators that tag proteins for degradation by the 26S proteasome (Bodine & Baehr, 2014).

In the present study, PG‐PS administration elevated 4‐HNE levels, a widely recognized marker of lipid peroxidation. Increased 4‐HNE levels indicate elevated oxidative stress, which directly impairs protein function and cellular integrity (Choi et al., 2016; Kasai et al., 2022). These oxidative modifications contribute to muscle protein breakdown and inhibit protein synthesis, accelerating muscle atrophy (Powers et al., 2010). However, the puromycin‐labeled proteins and mRNA expression of Atrogin‐1 and MuRF‐1 did not show the main effect of PG‐PS. These results indicate a discrepancy between morphological and molecular findings. This discrepancy suggests that temporal changes or the MPB pathway, which were not evaluated in this study (e.g., autophagy pathway, apoptosis), contribute to skeletal muscle atrophy. In future studies, we need to elucidate the detailed molecular mechanisms, including the different temporal dynamics.

### 4.4 Skeletal muscle adaptation by voluntary wheel running (VWR) following exposure to PG‐PS–induced CSI

VWR increased muscle weight and fiber CSA in both saline‐ and PG‐PS‐treated mice, indicating that these hypertrophic responses were preserved following PG‐PS exposure. A transient reduction in daily running distance was observed on day 1 in PG‐PS‐treated mice; however, total running distance was not different between groups. These findings suggest that the transient reduction in running behavior did not substantially influence overall training adaptations.

VWR did not alter myofibre type or mitochondria‐related protein expression. Overall, 3 weeks of VWR does not induce fiber type transitions or changes in mitochondrial function in the soleus muscle in mice (Egawa et al., 2022; Ikeda et al., 2006; Pellegrino et al., 2005), likely due to its inherently high oxidative capacity. More intense aerobic stimuli may be required to induce changes in mitochondrial content or fiber type.

In this study, VWR was associated with reduced markers of CSI and was not impaired by PG‐PS treatment, indicating that appropriate exercise modalities can support muscle adaptation even under inflammatory conditions. The exercise groups exhibited increased puromycin‐labeled proteins and elevated phosphorylation of p70S6K in the soleus muscle. In addition, when total and phosphorylated protein levels were analyzed separately, a main effect of exercise was observed in both total and phosphorylated rpS6 (see Supplementary Figure S1). These findings suggest that VWR‐induced hypertrophy involves activation of MPS via the mTORC1 signaling pathway, with increasing total rpS6 amount. This suggests that increases in ribosomal proteins might be involved in this phenomenon. Additionally, suppression of MPB in the exercise groups may have contributed to the hypertrophic response.

Muscle hypertrophy induced by electrically stimulated gastrocnemius contractions is limited under CSI; however, this impairment can be alleviated by combining resistance training with an anti‐inflammatory diet (Sumi et al., 2020). These findings underscore the importance of controlling inflammation for adequate training adaptation during CSI. Unlike electrical stimulation of localized muscles, this study employed VWR, a physiologically relevant whole‐body exercise model. Muscle contraction induces myokine secretion, including IL‐6 and IL‐15, which regulate inflammation by suppressing pro‐inflammatory cytokines and enhancing anti‐inflammatory as well as antioxidant responses (Pedersen et al., 2007; Li et al., 2014; Petersen & Pedersen, 2005). These myokines may have contributed to the reduced TNF‐α and IL‐1β levels observed in this study, which may be associated with lower systemic inflammation and support for muscle protein synthesis‐related signaling. However, Komine et al. reported that whole‐body exercise enhanced the clearance of inflammatory mediators such as LPS (Komine et al., 2017); therefore, further studies are required to elucidate the underlying mechanisms. Collectively, whole‐body exercise increased skeletal muscle mass and fiber size regardless of PG‐PS treatment and may represent a promising intervention for inflammation‐associated muscle wasting.

Future clinical research should identify optimal exercise prescriptions (e.g., intensity, frequency, and timing), examine the synergy between exercise and anti‐inflammatory therapies, and determine whether similar benefits occur across different patient populations. Such studies may contribute to the development of personalized rehabilitation strategies for preserving muscle health and functional independence in populations with chronic inflammation.

### 4.5 Limitations

This study has several limitations. First, although the PG‐PS model was used to induce CSI, it does not fully recapitulate the complexity of chronic disease conditions in humans. Therefore, future studies should employ more clinically relevant disease models. Second, the exercise intervention was initiated immediately after PG‐PS injection. As previous studies using PG‐PS‐induced skeletal muscle atrophy models have typically been conducted within a three‐week period, the intervention duration in the present study was set accordingly. Therefore, whether similar effects would be observed with different intervention timing or duration, or at different time points, remains unclear. Third, this study did not include female mice. Previously, we demonstrated that PG‐PS‐induced skeletal muscle atrophy showed sex differences. Therefore, to eliminate the influence of sex differences, the present study was conducted exclusively in male mice. However, whether similar findings would be observed in females remains unclear. Future studies should incorporate aged and ovariectomised mouse models to determine whether these findings are maintained under altered sex hormone conditions.

In summary, this study investigated whether VWR‐induced skeletal muscle adaptations are maintained following CSI exposure. PG‐PS administration increased systemic inflammation and was associated with lower muscle mass under sedentary conditions; however, VWR increased muscle mass and fiber size in both saline‐ and PG‐PS‐treated mice, indicating that exercise‐induced adaptations were not impaired by PG‐PS treatment. Clinically, these findings suggest that even under chronic inflammatory conditions, whole‐body exercise may preserve skeletal muscle mass. This is particularly relevant for patients with chronic inflammatory diseases, such as rheumatoid arthritis, inflammatory bowel disease, and chronic obstructive pulmonary disease, wherein low‐grade systemic inflammation along with inactivity accelerate sarcopenia and frailty. Exercise represents a non‐pharmacological, low‐cost, and widely accessible intervention. Notably, our data indicate that relatively mild aerobic activity is sufficient to suppress systemic inflammation and support muscle protein turnover, making this a feasible approach for patients with limited exercise capacity.

## AUTHOR CONTRIBUTIONS

**Ryoga Kinoshita:** Conceptualization; data curation; formal analysis; investigation; methodology; project administration; validation; visualization. **Tatsuhiro Yamaguchi:** Supervision. **Satoru Ato:** Supervision. **Karina Kouzaki:** Investigation; supervision. **Yuki Tamura:** Supervision. **Koichi Nakazato:** Conceptualization; funding acquisition; methodology; supervision.

## FUNDING INFORMATION

This research received no external funding.

## CONFLICT OF INTEREST STATEMENT

The authors declare no conflicts of interest, financial or otherwise.

## Supporting information


**Figure S1.** VWR increased p70 ribosomal S6 kinase (p70S6K) and ribosomal protein S6 (rpS6) phosphorylation and total rpS6. (a) Total p70S6K in the soleus muscle (Saline+Sed group, *n* = 12; PG‐PS + Sed group, *n* = 12; Saline+Ex group, *n* = 12; PG‐PS + Ex group, *n* = 12). (b) Total rpS6 in the soleus muscle (Saline+Sed group, *n* = 12; PG‐PS + Sed group, *n* = 12; Saline+Ex group, *n* = 12; PG‐PS + Ex group, *n* = 12). (c) Total 4E‐binding protein 1 (4E‐BP1) in the soleus muscle (Saline+Sed group, *n* = 12; PG‐PS + Sed group, *n* = 12; Saline+Ex group, *n* = 12; PG‐PS + Ex group, *n* = 12). (d) Phosphorylated p70S6K in the soleus muscle (Saline+Sed group, *n* = 12; PG‐PS + Sed group, *n* = 12; Saline+Ex group, *n* = 12; PG‐PS + Ex group, *n* = 12). (e) Phosphorylated rpS6 in the soleus muscle (Saline+Sed group, *n* = 12; PG‐PS + Sed group, *n* = 12; Saline+Ex group, *n* = 12; PG‐PS + Ex group, *n* = 12). (f) Phosphorylated 4E‐BP1 in the soleus muscle (Saline+Sed group, *n* = 12; PG‐PS + Sed group, *n* = 12; Saline+Ex group, n = 12; PG‐PS + Ex group, *n* = 12). Data are shown as mean ± SD. A two‐way ANOVA was performed.

## Data Availability

All data generated and analyzed in the present study are available from the corresponding author upon reasonable request.
