# Supplementary material for: Voluntary wheel running attenuates peptidoglycan–polysaccharide‐induced inflammation and preserves skeletal muscle remodeling in male C57BL/6J mice
Source: Physiol Rep. 2026 May 18;14(10):e70916. doi: 10.14814/phy2.70916 (PMC13183601; doi:10.14814/phy2.70916)
Supplement: Supplementary file 1 — Figure S1. VWR increased p70 ribosomal S6 kinase (p70S6K) and ribosomal protein S6 (rpS6) phosphorylation and total rpS6. (a) Total p70S6K in the soleus muscle (Saline+Sed group, n = 12; PG‐PS + Sed group, n = 12; Saline+Ex group, n = 12; PG‐PS + Ex group, n = 12). (b) Total rpS6 in the soleus muscle (Saline+Sed group, n = 12; PG‐PS + Sed group, n = 12; Saline+Ex group, n = 12; PG‐PS + Ex group, n = 12). (c) Total 4E‐binding protein 1 (4E‐BP1) in the soleus muscle (Saline+Sed group, n = 12; PG‐PS + Sed group, n = 12; Saline+Ex group, n = 12; PG‐PS + Ex group, n = 12). (d) Phosphorylated p70S6K in the soleus muscle (Saline+Sed group, n = 12; PG‐PS + Sed group, n = 12; Saline+Ex group, n = 12; PG‐PS + Ex group, n = 12). (e) Phosphorylated rpS6 in the soleus muscle (Saline+Sed group, n = 12; PG‐PS + Sed group, n = 12; Saline+Ex group, n = 12; PG‐PS + Ex group, n = 12). (f) Phosphorylated 4E‐BP1 in the soleus muscle (Saline+Sed group, n = 12; PG‐PS + Sed group, n = 12; Saline+Ex group, n = 12; PG‐PS + Ex group, n = 12). Data are shown as mean ± SD. A two‐way ANOVA was performed. [file PHY2-14-e70916-s001.docx]

Supplementary Fig. S1. VWR increased p70 ribosomal S6 kinase (p70S6K) and ribosomal protein S6 (rpS6) phosphorylation and total rpS6. (A) Total p70S6K in the soleus muscle (Saline+Sed group, n = 12; PG-PS+Sed group, n = 12; Saline+Ex group, n = 12; PG-PS+Ex group, n = 12). (B) Total rpS6 in the soleus muscle (Saline+Sed group, n = 12; PG-PS+Sed group, n = 12; Saline+Ex group, n = 12; PG-PS+Ex group, n = 12). (C) Total 4E-binding protein 1 (4E-BP1) in the soleus muscle (Saline+Sed group, n = 12; PG-PS+Sed group, n = 12; Saline+Ex group, n = 12; PG-PS+Ex group, n = 12). (D) Phosphorylated p70S6K in the soleus muscle (Saline+Sed group, n = 12; PG-PS+Sed group, n = 12; Saline+Ex group, n = 12; PG-PS+Ex group, n = 12). (E) Phosphorylated rpS6 in the soleus muscle (Saline+Sed group, n = 12; PG-PS+Sed group, n = 12; Saline+Ex group, n = 12; PG-PS+Ex group, n = 12). (F) Phosphorylated 4E-BP1 in the soleus muscle (Saline+Sed group, n = 12; PG-PS+Sed group, n = 12; Saline+Ex group, n = 12; PG-PS+Ex group, n = 12). Data are shown as mean ± SD. A two-way ANOVA was performed.
